# National variation in the composition of rheumatology multidisciplinary teams: a cross-sectional study

**DOI:** 10.1007/s00296-017-3751-0

**Published:** 2017-05-27

**Authors:** Mwidimi Ndosi, Rachel Ferguson, Michael R. Backhouse, Lindsay Bearne, Phillip Ainsworth, Alan Roach, Elaine Dennison, Lindsey Cherry

**Affiliations:** 1 Department of Nursing and Midwifery, Centre for Health and Clinical Research, University of the West of England, Bristol, UK; 2 Academic Rheumatology Unit, Bristol Royal Infirmary, Bristol, UK; 3 Faculty of Health Sciences, University of Southampton, Southampton, UK; 4 Department of Podiatry, Solent NHS Trust, Southampton, UK; 5 Leeds Institute of Rheumatic and Musculoskeletal Medicine, University of Leeds, Leeds, UK; 6 NIHR Leeds Biomedical Research Unit, Leeds Teaching Hospitals NHS Trust, Leeds, UK; 7 Division of Health and Social Care Research, King’s College London, London, UK; 8 Suffolk Agricultural Association, Suffolk, UK; 9 British Society for Rheumatology, London, UK; 10 MRC Lifecourse Epidemiology Unit, Southampton, UK

**Keywords:** Multidisciplinary, National survey, Arthritis, Health service, Rehabilitation, Team care

## Abstract

The objective of this study is to describe the composition of multidisciplinary teams (MDT) working within rheumatology departments across the UK. All rheumatology departments in the United Kingdom (UK) were invited to participate in a national electronic survey between February 2014 and April 2015 as a part of a national audit for the management of rheumatoid and early inflammatory arthritis commissioned by Healthcare Quality Improvement Partnership. Rheumatology departments were asked to report their MDT composition; defined as a rheumatologist (consultant or specialist trainee), specialist nurse, occupational therapist physiotherapist, and podiatrist. The data were collected as Whole Time Equivalent (WTE) of each professional group at each department adjusted to 100,000 population. The data were grouped according to British Society for Rheumatology regions to study regional variations. The survey was completed by 164/167 departments (98% response rate). All departments reported an MDT comprising a rheumatologist (consultant or specialist trainee) and almost all included a specialist nurse but only 28 (17%) of the departments had MDTs comprising all the professional groups. There was a high degree of regional variation in the provision of Allied Health Professionals (physiotherapists, occupational therapists, and podiatrists) in the UK. MDT care is recommended for the management of inflammatory arthritis, but few UK rheumatology departments have a full complement of healthcare professionals within their MDT. There is a high degree of regional variation in the composition and staffing levels of the rheumatology MDT across the UK; the impact of which warrants further investigation.

## Introduction

The last two decades have seen dramatic developments in the management of rheumatic and musculoskeletal diseases (RMDs) mainly due to improvements in the diagnostic techniques, treatment strategies, and outcome measurement. Patient care has shifted from a mainly in-patient to outpatient model, where the patients self-manage some aspects of their disease and have access to support from a diverse group of health professionals forming the multidisciplinary team (MDT). This model of care is considered to represent the best clinical practice and is recommended by the current treatment guidelines for inflammatory arthritis (IA) [1–3] and other long-term conditions [4–6].

MDT working can be defined as members of different health care professions with specialised skills and expertise working together to support people with complex care needs [7]. In rheumatology services, the composition of the MDT would normally include a rheumatologist (a consultant and/or a specialist registrar), a specialist nurse, a physiotherapist, an occupational therapist, and a podiatrist [3, 6, 8]. However, there is a lack of consensus about the optimal configuration of the MDT in rheumatology services.

A recent meta-review investigating the effectiveness of MDT care in other long-term conditions (CHF, Diabetes, COPD, and asthma) demonstrated benefits in clinical, functional, and patient-centred outcomes [9]. While some aspects of patient outcomes reported in the meta-review are important in rheumatology (improved function, quality of life, satisfaction with care, adherence to therapy, reduced readmissions, and mortality), the effectiveness of MDT working in rheumatology is unclear. Whilst there is evidence to support the effectiveness of single disciplines in the management of specific patient groups [10–12], a systematic review of effectiveness of MDT care found limited evidence on disability, disease activity, or quality of life in people with rheumatoid arthritis (RA) [13]. The coordination of MDT care seems to be the key to its effectiveness [14]. Teams can be said to work at an ‘interdisciplinary’ level if working in a highly coordinated way with all team members working towards shared goals [14]. However, in the United Kingdom (UK), the composition of MDT in rheumatology is unknown and understanding the composition is important if interdisciplinary care is to be achieved.

The British Society for Rheumatology (BSR) conducted two national audits [15, 16] to assess the services available to patients when referred to rheumatology units with suspected early inflammatory arthritis (IA). The audits were commissioned by the Healthcare Quality Improvement Partnership (HQIP) as part of the National Clinical Audit Programme. The first audit was conducted between February 2014 and January 2015 and the second one between February 2015 and January 2016. We carried out an analysis of the first audit data with additional data from the UK devolved nations, to study the composition of MDT within rheumatology departments in the UK.

## Methods

### Design

This was a cross-sectional descriptive study conducted by survey in all rheumatology departments within the UK. In England, the survey was a part of a broader national audit for rheumatoid and early inflammatory arthritis, commissioned by HQIP [15]. As Scotland, Northern Ireland and the Channel Islands were not included in the HQIP audit, a separate but identical, service survey of all rheumatology departments was conducted. Ethical approval was not required, but access to the data was granted by HQIP and supported by the BSR Research Committee and British Health Professionals in Rheumatology (BHPR).

### Development of the survey content

A project working group was convened to design the survey content. This comprised senior clinicians and academics from several UK institutions, representatives from partner organisations and patient groups, working collaboratively on behalf of the BSR and BHPR [15, 16]. The survey included organisation data regarding the specific inclusion of, or direct access to, a rheumatologist, a specialist nurse, a physiotherapist, a podiatrist, and an occupational therapist as part of the MDT, including detail of their whole time equivalent (WTE) availability.

### Data collection

Northgate Public Services, a software and outsourcing business, provided secure online databases and electronic audit tools which were made available to all rheumatology units. Clinicians or administrators at each department uploaded their data securely onto the online database and the transferred to the MRC Lifecourse Epidemiology Unit, University of Southampton for analysis.

### Statistical analysis

The staffing levels were measured in numbers of whole time equivalent (WTE) for each professional group. The data were analysed descriptively using STATA version 12.1 for Windows, and summarised to determine the adjusted mean WTE and percentage of representation of each professional group per 100,000-catchment population. Further grouping of the data according to BSR regions was used to show regional variations.

## Results

The survey response rate was 98% with 164 out of 167 UK departments completing the survey. All MDTs managing IA include a physician (consultants and specialist trainees) and almost all include a specialist nurse. However, other allied health professional groups are not represented in all departments. For example, podiatrists are only available in 48% of MDTs. Of the 164 surveyed departments, only 28 (17%) had access to a full MDT including a rheumatologist, a specialist nurse, a physiotherapist, an occupational therapist, and a podiatrist. The adjusted mean WTE per 100,000 population ranged from 0.04 to 0.44 for rheumatologists, 0.02–0.15 for rheumatology trainees, 0.05–0.44 for specialist nurses, 0–0.7 for physiotherapists, 0.02–0.15 for occupational therapists, and 0–0.04 for podiatrists (see Table 1).

**Table 1:** Overall rheumatology MDT staffing levels between professional groups

Profession	Adjusted mean WTE^a^	SD	Range	Represented within MDT Y/N (%)
Consultants	0.08	1.64	0.04–0.44	164 (100)
Specialist trainee	0.02	0.28	0.02–0.15	132 (80)
Specialist nurses	0.08	1.89	0.05–0.44	162 (99)
Physiotherapists	0.03	0.85	0.00–0.70	120 (73)
Occupational therapists	0.03	0.85	0.02–0.15	123 (75)
Podiatrists	0.02	0.40	0.00–0.04	79 (48)

^a^Adjusted per 100,000 population

**Fig. 1:**
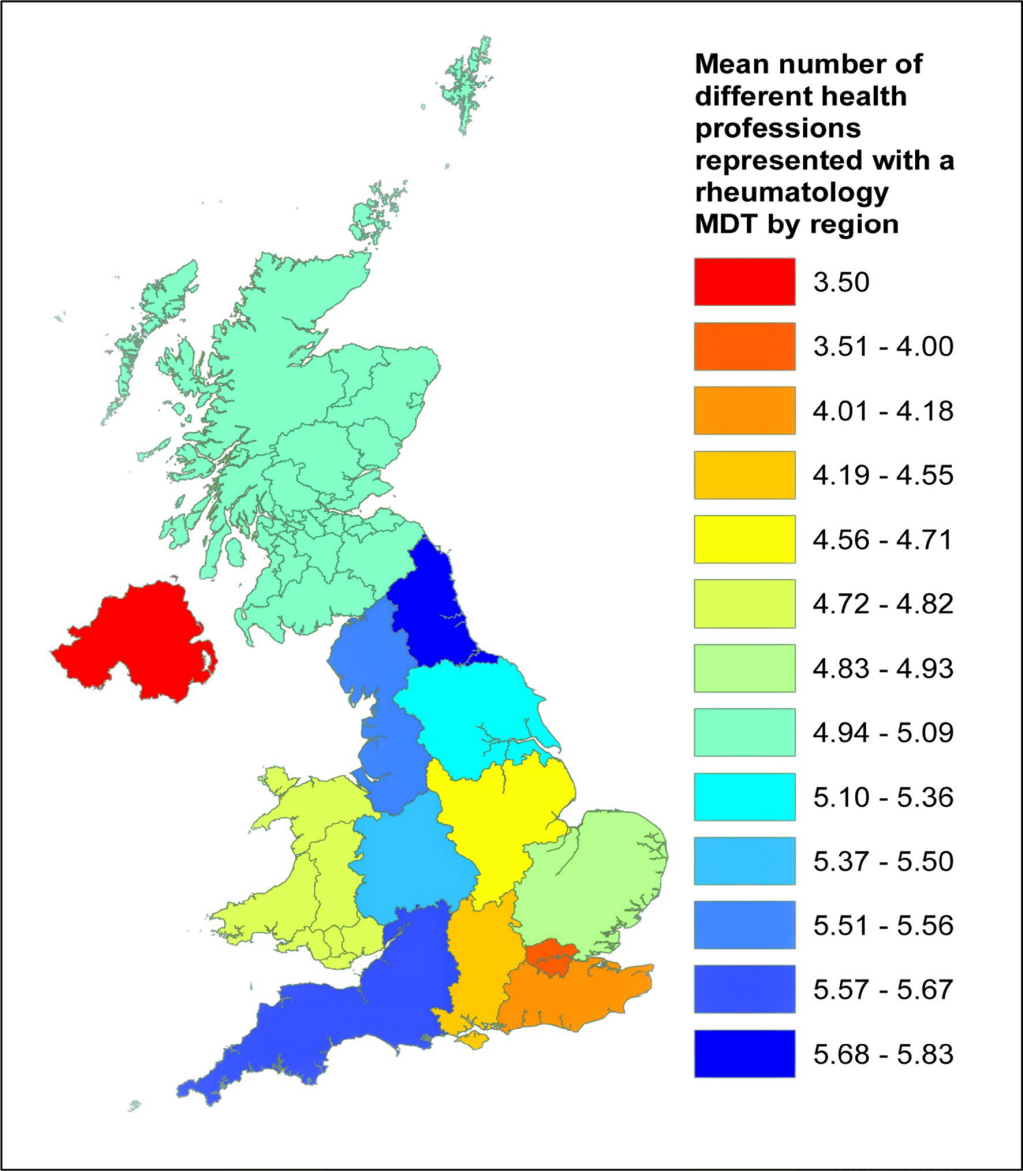
Choropleth map showing the mean number of different health professions represented within a rheumatology department MDT by region

Variation in the adjusted mean WTE availability of each professional group was notable when the BSR regions were considered. For example, the Northern Ireland had the highest adjusted mean WTE for rheumatologists, while London region had the lowest. For nurse specialists, Northern Ireland again had the highest adjusted mean WTE, while Scotland, London, South West and Yorkshire, and the Humber shared the lowest (Table 2). Northern Ireland had the lowest adjusted mean WTE for physiotherapists and the podiatrists (adjusted mean WTE for both professional groups was zero). These regional variations were evident across all professional groups and had no particular pattern. Figure 1 shows the regional variation in the (unadjusted) mean WTE staff levels across the UK.

**Table 2:** Variation in UK rheumatology staffing levels per region; values are reported as absolute mean and adjusted to per 100,000 population to account for variation in population size serviced by each region

Region	Number of departments	Total number of WTE staff	Mean WTE of all staff	Mean (and adjusted mean) WTE of individual professional groups						
					Consultants	Specialist trainees	Nurse Specialists	Physiotherapists	Occupational therapists	Podiatrists
East Midlands	7	116.02	4.71	Mean	3.91	1.36	7.97	2.34	1.52	1.50
				Adjusted mean	0.11	0.04	0.21	0.06	0.04	0.04
East of England	14	142.76	4.93	Mean	3.26	1.61	2.43	1.24	1.28	1.54
				Adjusted mean	0.24	0.04	0.06	0.03	0.03	0.04
London	23	290.74	4.00	Mean	3.87	1.71	4.75	3.09	2.14	0.74
				Adjusted mean	0.04	0.02	0.05	0.03	0.02	0.01
North East	6	123.53	5.83	Mean	5.06	1.50	4.27	1.42	1.41	0.78
				Adjusted mean	0.19	0.06	0.16	0.05	0.05	0.03
Northern Ireland	2	15.00	3.50	Mean	3.00	1.00	3.00	0.00	1.00	0.00
				Adjusted mean	0.44	0.15	0.44	0.00	0.15	0.00
North West	17	157.77	5.69	Mean	3.11	1.16	3.39	1.76	1.40	1.10
				Adjusted mean	0.05	0.02	0.06	0.03	0.02	0.02
Mersey	8	67.30	5.58	Mean	2.55	0.94	2.52	1.43	1.10	0.23
				Adjusted mean	0.10	0.04	0.10	0.06	0.04	0.01
Scotland	11	101.19	5.09	Mean	3.32	0.87	2.05	1.24	1.17	0.72
				Adjusted mean	0.07	0.02	0.05	0.03	0.03	0.02
South East	11	159.90	4.18	Mean	2.95	0.91	6.50	3.20	4.00	0.75
				Adjusted mean	0.06	0.02	0.14	0.07	0.09	0.02
South Central	11	102.24	4.55	Mean	3.30	1.31	3.05	1.29	0.84	0.98
				Adjusted mean	0.08	0.03	0.08	0.03	0.02	0.02
South West	15	139.86	5.67	Mean	2.95	0.97	2.46	1.18	1.20	0.67
				Adjusted mean	0.06	0.02	0.05	0.02	0.02	0.01
Yorkshire and the Humber	14	183.45	5.36	Mean	4.09	1.33	2.98	1.61	2.25	0.99
				Adjusted mean	0.06	0.02	0.05	0.03	0.04	0.02
Wales	11	175.30	4.82	Mean	9.02	0.91	2.68	2.12	1.15	1.05
				Adjusted mean	0.28	0.03	0.08	0.07	0.04	0.03
West Midlands	14	204.30	5.50	Mean	4.19	1.24	6.45	1.36	0.94	0.50
				Adjusted mean	0.07	0.02	0.11	0.02	0.02	0.01

## Discussion

The findings of this national survey provide recent information regarding the inclusion of the five professional groups in rheumatology MDT. Despite being the cornerstone of the management of IA [3, 6], MDT provision in the UK is variable and, at times, only reaches the minimum definition for MDT care.

Our results show that all rheumatology departments have an MDT which comprises a rheumatologist and almost all have access to a nurse specialist but the inclusion of other allied healthcare professional groups is variable and podiatrists, in particular, are poorly represented. Only 17% of the surveyed departments meet the current national guidance [3, 6] by having the five professional groups represented in their MDTs.

Our data clearly demonstrate that access to the three professional groups (physiotherapists, occupational therapists, and podiatrists) is inadequate. Patients access these professional groups via three main routes: a referral by the general practitioner (GP), the rheumatologist (consultant), or hospital in-patient services. In 2009, an audit of acute trusts found that only 73% of acute trusts provided access to physiotherapists, 64% to occupational therapist, and 55 to podiatrists [17]. For physiotherapy, a patient survey in 2011 [18] revealed that 31% of patients had never been referred for physiotherapy. Among those who were referred, 32.2% waited for over 1 year to see a physiotherapist. Our data suggest that there was no improvement in the access to physiotherapists over four years and a little improvement in the access to occupational therapists. Our survey suggests that access to podiatrists by patients with RA is improving but is still poor despite national guidance [3, 6]. Previously, both an inception cohort [19] and the national survey [8] found that between 28 and 30% of patients with RA had access to a podiatrist.

While 99% of MDTs in our data have nurse specialist representation, we do not know if each centre has sufficient specialist nurses to meet the needs of patients. This is particularly important as the nursing staffing levels are linked with patient outcomes especially those related to initiation and escalation of treatments and monitoring of disease activity [10, 16]. However, we do not know the optimum staffing levels required to maximise patient benefit and this is an area for further research.

The high degree of regional variation in the provision of allied health professional services highlights the absence of some specialist services, such as physiotherapy, occupational therapy, and podiatry, in some departments. For example, the two centres surveyed in Northern Ireland reported no access to a physiotherapist or podiatrist. In the national survey conducted in 2006 [8], Northern Ireland also reported no access to podiatry, which is concerning as there has been little change in service provision over the last decade, despite the publication of national management guidelines.

Identifying how MDTs meet the care needs of patients was beyond the scope of this study, but the regional variations and unavailability of some MDT services may have implications to patients’ care and outcomes. The natural progression in IA is a decline in function and the evidence from several long-term conditions suggests that optimising MDT care promotes rehabilitation [14]. Inequitable access to MDT care could mean that some patients might be referred to general physiotherapy, occupational therapy, or podiatry services, which may not have specialist rheumatology knowledge. This could delay patient access to specialist management and affect patient outcomes and productivity. Our findings suggest that UK rheumatology MDT composition may be more variable than in other Northern European countries. The study conducted by the Scandinavian Team Arthritis Register—European Team Initiative for Care Research (STAR-ETIC) collaboration [20] revealed large similarities in the composition of MDT teams across four Northern European countries (Sweden, The Netherlands, Denmark and Norway). Nine out of the 10 Rheumatology centres investigated included a rheumatologist, a nurse, a physiotherapist, an occupational therapist and a social worker in their MDTs, although provision of podiatrists, psychologists, and nutritionists varied [20]. However, the STAR-ETIC study [20] did not report the national picture of MDT provision in these countries therefore, whilst their findings are interesting, they are unlikely to be representative of MDT provision in Northern Europe. The UK national guidance recommends access to MDT [6] and our data provide good evidence of the extent to which this standard has been achieved nationally. Efforts can now be directed towards addressing inequitable access to the MDT.

Our study has two main limitations. First, our data provide only cross-sectional information on the availability of the professionals included in the rheumatology MDT within the UK. However, this information will be useful and act as a baseline for future studies. Second, our data do not inform the level of coordination or the interaction of the members within the MDTs. The national guidelines [3, 6] do not specify the proportion of professional representation or the level of coordination within the MDT. This study has determined the composition of the MDTs and future research is required to determine the optimal configuration and interaction of rheumatology MDT to inform practice and policy.

In conclusion, this study shows that over three-quarters of rheumatology teams in the UK do not have all recommended professional groups represented in their MDTs thus fall short of the quality standards of care for people with IA. There is a high degree of regional variation in the composition and staffing levels of the rheumatology MDT and future studies should investigate the impact of these variations. Efforts should be directed towards improving equitable access to rheumatology specialist services to optimise outcomes for people with IA.
